# Evaluation of R2CHADS2, R2CHA2DS2-VASc, and R2CHA2DS2-VA Scores for the Prediction of In-Hospital Mortality in Patients with ST-Elevation Myocardial Infarction

**DOI:** 10.3390/jcm14134624

**Published:** 2025-06-30

**Authors:** Evliya Akdeniz, Cennet Yıldız, Mehmet Pisirici, Hasan Ali Sinoplu, Dilay Karabulut, Fatma Nihan Turhan Çağlar

**Affiliations:** Department of Cardiology, Bakirkoy Dr. Sadi Konuk Training and Research Hospital, Istanbul 34147, Turkey; evliyakdeniz@gmail.com (E.A.); pisiricimehmet@gmail.com (M.P.); hasanalisinoplu@gmail.com (H.A.S.); dilay_karakozak@hotmail.com (D.K.); nhnturhan@gmail.com (F.N.T.Ç.)

**Keywords:** ST-elevation myocardial infarction, mortality, kidney, R2CHADS2, R2CHA2DS2-VASc, R2CHA2DS2-VA

## Abstract

**Background/Objectives**: Despite the contemporary management of ST segment elevation myocardial infarction (STEMI) patients, in-hospital mortality rates remain considerable. Therefore, the assessment of in-hospital mortality risk of patients with STEMI has a major role in terms of disease course. R2CHADS2, R2CHA2DS2-VASc, and R2CHA2DS2-VA scores are potential candidate for the prediction of in-hospital mortality in STEMI patients. This study aims to determine the association between R2CHADS2, R2CHA2DS2-VASc, and R2CHA2DS2-VA scores and in-hospital mortality in patients with STEMI who have undergone primary percutaneous coronary intervention (p-PCI). **Methods**: A total of 857 consecutive patients diagnosed with STEMI who were admitted to our hospital and treated with p-PCI were included in our study. **Results**: The mean age of the study population was 58 ± 11 years and the population was predominantly male (78.5%). Patients in the in-hospital mortality group tended to be older compared to those who survived (65 ± 12 and 57 ± 11 years, respectively, *p* < 0.001), while gender showed no significant difference. Multivariable regression models showed that left ventricular ejection fraction, eGFR, R2CHADS2 (OR 2.21, 95% CI 1.38–3.54, *p* = 0.001), R2CHA2DS2-VASc (OR 1.91, 95% CI 1.30–2.80, *p* = 0.001), and R2CHA2DS2-VA (OR 1.97, 95% CI 1.345–2.910, *p* = 0.001) scores were independent predictors of in-hospital mortality. **Conclusions**: The R2CHADS2, R2CHA2DS2-VASc, and R2CHA2DS2-VA scores demonstrate strong predictive ability for in-hospital mortality in STEMI patients, and their non-negligible advantages support their implementation in clinical practice.

## 1. Introduction

Although the contemporary treatment method for patients with ST segment elevation myocardial infarction (STEMI) is primary percutaneous coronary intervention (p-PCI), the in-hospital mortality rates can rise up to 9.2%, varying from region to region [1,2,3]. In daily cardiology practice, given its pivotal role for the management and survival of STEMI patients, it is crucial to identify high-risk patients in terms of in-hospital mortality. Accurate risk assessment aids in the effective management of patients and improves the overall efficiency of the healthcare system. Therefore, the European Society of Cardiology (ESC) recommends the early risk assessment of STEMI patients [4]. Although the use of the Global Registry of Acute Coronary Events (GRACE) score for clinical risk assessment is recommended, it is important to note that the GRACE score is a prediction model developed based on a study including patients with STEMI and non-ST elevation acute coronary syndrome [5].

To date, many predictors have been identified with the aim of assessing the adverse clinical event risk in patients with acute myocardial infarction. These predictors can include a range of variables, such as demographic or clinical characteristics, laboratory parameters, and echocardiographic and angiographic features of the patients [6,7,8,9].

The CHA2DS2-VASc scoring system, which can be described as an advanced version of the CHADS2 score, is derived from such variables as congestive heart failure, hypertension, age, diabetes, stroke, vascular diseases, and gender. It is used to determine the risk of stroke in patients with atrial fibrillation (AF). The CHADS2 score itself is based on variables including congestive heart failure, hypertension, age, diabetes, and stroke [10,11]. The R2CHADS2 score is an extension of the CHADS2 score, adding 2 points if renal dysfunction is present [12]. Since its initial introduction, the CHA2DS2-VASc score has been shown not only to predict stroke risk but also to be useful in prediction survival in various clinical scenarios, such as AF, infective endocarditis, and acute coronary syndrome [13,14,15].

After discovering the predictive power of the CHA2DS2-VASc score, a novel mortality predictor was proposed by incorporating renal function into the score, with the acronym R2CHA2DS2-VASc. The R2CHA2DS2-VASc score has an effective capability for predicting mortality in various clinical scenarios, such as acute coronary syndrome patients, high cardiovascular risk populations, or patients undergoing percutaneous transcatheter aortic valve replacement [16,17,18]. However, the evidence of importance of the R2CHA2DS2-VASc score in determining the risk of in-hospital mortality for STEMI patients treated with p-PCI is lacking. In the latest guidelines for AF management by the ESC, it is now recommended to use the CHA2DS2-VA score instead of the traditional CHA2DS2-VASc score [19]. Accordingly, we integrated renal function into the CHA2DS2-VA score to develop the R2CHA2DS2-VA score and assessed its predictive performance for in-hospital mortality among patients with STEMI.

In this study, our goal is to investigate the association between R2CHADS2, R2CHA2DS2-VASc, and R2CHA2DS2-VA scores and in-hospital mortality in patients with STEMI who have undergone p-PCI.

## 2. Materials and Methods

### 2.1. Study Population

Between December 2022 and August 2024, consecutive patients with a diagnosis of STEMI who underwent p-PCI at our center were included in the study. The inclusion criteria were age to be older than 18 years, to have an ST segment elevation for at least two consecutive leads on a surface electrocardiogram, and to be treated with p-PCI. Exclusion criteria were the patient’s refusal to participate in the study, failed p-PCI, terminal stage oncologic disorders, and end stage liver disease. Baseline demographic features and clinical and laboratory variables were obtained from hospital information system and national digital health system. Left ventricle ejection fraction (LVEF) was calculated via transthoracic echocardiography by using the modified Simpson method after the p-PCI procedure while the patients were in coronary intensive care unit.

### 2.2. Calculations

The CHADS2 score is based on variables including congestive heart failure, hypertension, age, diabetes, and stroke. One point each is given for congestive heart failure, hypertension, an age of 75 years or older, diabetes, while two points are given for a history of stroke or transient ischemic attack [10,11]. The R2CHADS2 score is an extension of the CHADS2 score, adding 2 points if renal dysfunction is present [12].

The CHA2DS2-VASc score is calculated by assigning points as follows: 1 point each is given for heart failure, hypertension, and age of 65 years or older, diabetes, vascular disease, and female gender; two points each are given for an age of 75 years or older and a history of stroke or transient ischemic attack. The total score is then summed to determine the risk [11]. The R2CHA2DS2-VASc score is provided by adding 2 points to the CHA2DS2-VASc score for patients with a glomerular filtration rate (GFR) ≤ 60 mL/min/1.73 m^2^, which was calculated with CKD-EPI formula [20].

### 2.3. Statistical Analysis

Normality of the data was assessed using Kolmogorov–Smirnov test. Continuous data were expressed as the mean and standard deviation and categorical data were expressed as numbers and percentages. Two-group comparisons were performed using the independent samples *t*-test or Mann–Whitney U test depending on the distribution of the data. Categorical variables were compared using the chi-square test. Receiver operating characteristic (ROC) curve analysis was used to determine the cut-off values of CHADS2, R2CHADS2, CHA2DS2-VASc, R2CHA2DS2-VASc, CHA2DS2-VA, and R2CHA2DS2-VA scores for predicting in-hospital mortality. Univariate logistic regression analysis was performed to find the independent predictors of in-hospital mortality. The parameters that were found to be significant in univariate logistic regression analysis were entered into multivariate logistic regression analysis. We conducted three multivariate logistic regression analyses, and each of them had two models. The first analysis compared the CHADS2 and R2CHADS2 scores in MODEL A and MODEL B, respectively, the second analysis compared the CHA2DS2-VASc and R2CHA2DS2-VASc scores in MODEL A and MODEL B, respectively, and the third analysis compared the CHA2DS2-VA and R2CHA2DS2-VA scores in MODEL A and MODEL B, respectively. Calibration of the models was assessed using the Hosmer–Lemeshow test and calibration plots, which compared the predicted probabilities to the observed (O) versus expected (E) proportions. SPSS version 25 statistical software (IBM Corp., Armonk, NY, USA) was used in the application of statistical analysis. *p* < 0.05 was considered statistically significant.

### 2.4. Endpoint

In-hospital mortality was the primary endpoint of our study.

## 3. Results

After excluding patients who did not meet the inclusion criteria for our study, a total of 857 consecutive patients diagnosed with STEMI who were admitted to our hospital and treated with p-PCI were included. The mean age of study population was 58 ± 11 years, and the population was predominantly male, with female patients representing 21.4% (n = 184) of the total. No significant difference was observed between the gender. Hypertension was present in 45.1% (n = 387) of the total population and diabetes mellitus was observed in 28.2% (n = 242). Patients in the in-hospital mortality group tended to be older compared to those who survived (65 ± 12 and 57 ± 11 years, respectively, *p* < 0.001). On the other hand, the mean of EF values were found to be lower in the in-hospital mortality group than those in the control group (29 ± 9% and 46 ± 10%, respectively, *p* < 0.001). Patients in the in-hospital mortality group had lower blood pressure, higher heart rates, lower eGFR, and higher serum creatinine, troponin, and natriuretic peptide levels than those in the control group. In addition, the lack of TIMI 3 epicardial blood flow was more common in the group of in-hospital mortality (25%) than those in the control group (10.9%), a difference that reached statistical significance (*p* = 0.010). The incidence of multivessel coronary disease was markedly higher in the in-hospital mortality group compared to the control group (43.2% vs. 23.5%, *p* = 0.005). CHADS2, R2CHADS2, CHA2DS2-VASc, R2CHA2DS2-VASc, CHA2DS2-VA and R2CHA2DS2-VA scores were all significantly higher in the in-hospital mortality group compared to those who survived. The baseline clinical characteristics for both groups are presented in Table 1.

In the univariate analysis (Table 2) age, body mass index, EF, blood pressure, heart rate, history of congestive heart failure, diabetes, serum albumin, C-reactive protein and glucose levels, and eGFR, CHADS2, CHA2DS2-VASc, CHA2DS2-VA, R2CHADS2, R2CHA2DS2-VASc, and R2CHA2DS2-VA scores were determined as risk factors of in-hospital mortality in STEMI patients. When multivariable regression models (Table 3A–C) are conducted based on these parameters, it has been determined that LVEF, eGFR, R2CHADS2 score (OR 2.21, 95% CI 1.38–3.54, *p* = 0.001), R2CHA2DS2-VASc score (OR 1.91, 95% CI 1.30–2.80, *p* = 0.001), and R2CHA2DS2-VA (OR 1.97, 95% CI 1.345–2.910, *p* = 0.001) score are independent predictors of in-hospital mortality. It should be addressed that although the CHADS2, CHA2DS2-VASc, and CHA2DS2-VA scores themselves are not significantly associated with in-hospital mortality, the R2CHADS2, R2CHA2DS2-VASc and R2CHA2DS2-VA scores obtained by merging eGFR with the CHADS2, CHA2DS2-VASc, and CHA2DS2-VA scores are the strongest predictors of in-hospital mortality.

**Figure 1:**
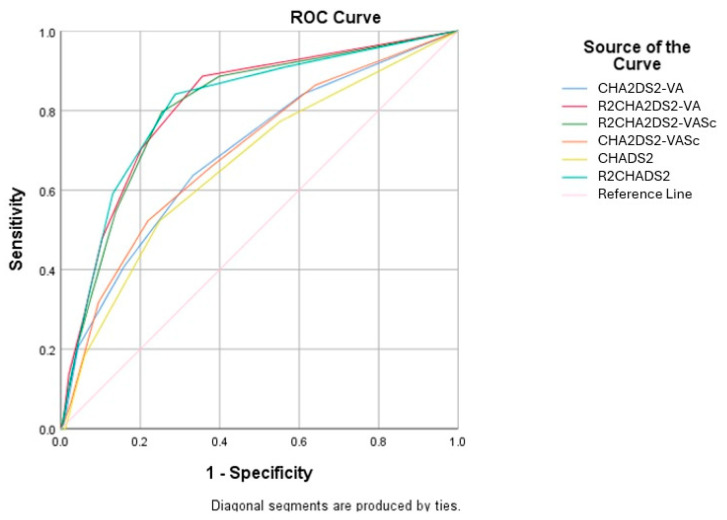
Receiver operating characteristic (ROC) curve analysis of CHADS2, R2CHADS2, CHA2DS2-VASc, and R2CHA2DS2-VASc scores for predicting in-hospital mortality.

As illustrated in Figure 1, in the receiver operating characteristic (ROC) curve, the cut-off values of R2CHADS2, R2CHA2DS2-VASc, and R2CHA2DS2-VA scores for the prediction of in-hospital mortality are ≥1.5 and ≥2.5, respectively. The determined cut-off values for the R2CHADS2, R2CHA2DS2-VASc, and R2CHA2DS2-VA scores predict in-hospital mortality with sensitivities of 84.1%, 79.5%, and 70.5% and specificities of 71.2%, 74.7%, and 79.7%, respectively. The area under curve (AUC) of R2CHADS2 score was calculated to be 0.810 (95% CI, 0.744–0.877, *p* < 0.001), that of R2CHA2DS2-VASc was calculated to be 0.807 (95% CI, 0.742–0.872, *p* < 0.001) and that of R2CHA2DS2-VA was calculated to be 0.814 (95% CI, 0.751–0.878, *p* < 0.001) (Table 4).

In addition, the study population was divided into two separate groups for R2CHADS, R2CHA2DS2-VASc, and R2CHA2DS2-VA scores, with each analysis consisting of two groups (Table 5). In the patient group with an R2CHADS score above 2, the in-hospital mortality rate was 19.7%, whereas in those with a score of 2 or below, this rate was only 2.5%. Similarly, in patients with a R2CHA2DS2-VASc score above 3, the in-hospital mortality rate was 17.6%, while in those with a score of 3 or below, it was 2.8%. Patients with an R_2_CHA_2_DS_2_-VA score of >3 experienced significantly higher in-hospital mortality (20%) compared to those with a score of ≤3 (3.5%).

**Figure 2:**
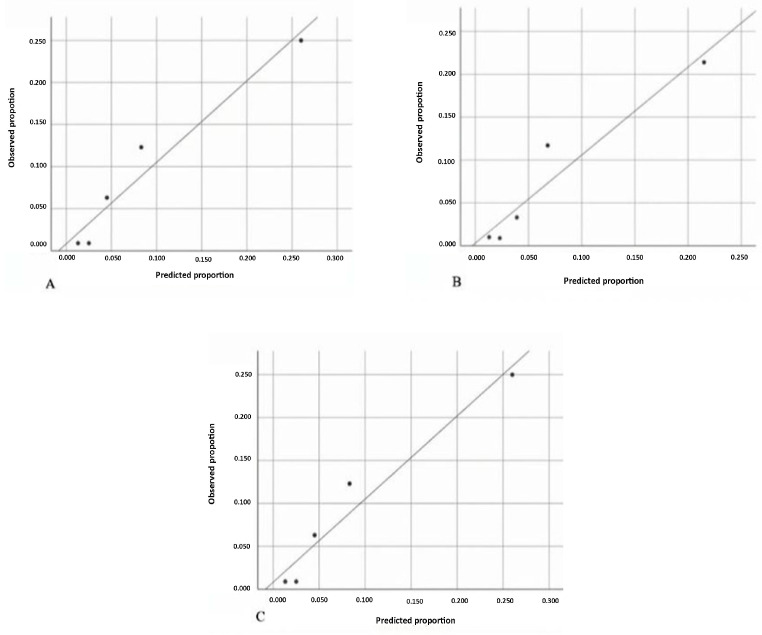
Calibration plots of the R2CHADS2, R2CHA2DS2-VASc, and R2CHA2DS2-VA scores for predicting in-hospital mortality. (**A**) R2CHADS2, (**B**) R2CHA2DS2-VASc, (**C**) R2CHA2DS2-VA spot: observed (proportion) line: predicted (proportion)

The Hosmer–Lemeshow test analysis demonstrated that the R2CHADS2, R2CHA2DS2-VASc, and R2CHA2DS2-VA scores showed good calibration for predicting in-hospital mortality, with *p*-values of 0.159, 0.188, and 0.187, respectively. The Hosmer–Lemeshow test results for the different scoring systems are summarized in Table 6. The observed and predicted proportions for each score are presented in Figure 2.

## 4. Discussion

This study aimed to evaluate the prognostic utility of the renal-adjusted risk scores, R2CHADS2, R2CHA2DS2-VASc, and R2CHA2DS2-VA, for predicting in-hospital mortality in patients with STEMI who underwent primary p-PCI. The most striking finding from our study was that the R2CHADS2, R2CHA2DS2-VASc, and R2CHA2DS2-VA scores were independent predictors of in-hospital mortality in STEMI patients treated with primary PCI. R2CHADS2, R2CHA2DS2-VASc, and R2CHA2DS2-VA scores were found to carry a 2.2-, 1.9-, and 1.9-fold risk of in-hospital mortality, respectively. In clinical practice, a R2CHADS2 score of 2 or more, a R2CHA2DS2-VASc score of 3 or more, and a R2CHA2DS2-VA score of 3 or more seem to be very useful tools to determine the high risk of in-hospital mortality in STEMI patients treated with p-PCI.

Although interventional procedures are commonly used in the treatment of myocardial infarction in contemporary cardiology practice, in-hospital mortality rates can still be considered as relatively high [1]. Therefore, early assessment of in-hospital mortality in STEMI patients is of great importance. Due to its paramount importance, there are numerous studies investigating predictors of in-hospital mortality in myocardial infarction patients during the p-PCI era. Chebab et al. demonstrated that >65 years of age, congestive heart failure, ejection fraction <35%, and a history of stroke were independent predictors of in-hospital mortality in STEMI patients. Since some clinical features used to derive the R2CHADS2 and R2CHA2DS2-VASc scores have been shown to be related to in-hospital mortality in this study, the results of our study appear to be logical and consistent [21]. The effect of female gender, another component of the R2CHADS2 and R2CHA2DS2-VASc score, on in-hospital mortality in STEMI patients has been investigated in the Belgian STEMI registry study. In this study, it was concluded that the in-hospital mortality risk for women is approximately 50% higher compared with men [3]. Qamar et al. demonstrated that lack of interventional treatment, female gender, diabetes, smoking, and age were independent factors negatively affecting in-hospital mortality in the NORIN-STEMI registry study [22]. Renal function emerges as another important clinical characteristic that needs to be emphasized in relation to this topic. The ANIN registry study demonstrated that, even with normal serum creatinine levels, lower GFR was associated with worse outcomes in terms of angiographic success and in-hospital adverse events. Therefore, it was concluded that GFR should be estimated in every STEMI patient [23]. The SWEDEHEART registry study also found an inverse relationship between renal function and in-hospital mortality. This relationship is similar in both STEMI and non-STEMI patients [24]. When we consider all this data together, the statistically significant relationship between the R2CHADS2 and R2CHA2DS2-VASc scores and in-hospital mortality is consistent with the scientific evidence.

The R2CHADS2 and R2CHA2DS2-VASc scores not only predict in-hospital mortality in STEMI patients but also provide valuable information about risk assessment in various clinical scenarios within the field of cardiology. For instance, in patients with systolic heart failure, the R2CHADS2 score seems to be more effective in predicting all-cause mortality than both the CHADS2 and CHA2DS2-VASc scores [25]. In addition, another observational study has shown that the R2CHADS2 score has prognostic significance in coronary artery disease patients [26]. Kiliszek et al. showed that the R2CHA2DS2-VASc score is associated with the long-term mortality of patients with acute coronary syndrome, including those with STEMI. Our study, which has been concluded that the R2CHA2DS2-VASc score predicts in-hospital mortality in STEMI patients, is complementary to these findings [17]. Beyond coronary heart disease, R2CHA2DS2-VASc score may be related to the mid- and long-term mortality of patients who have received transcatheter aortic valve replacement [18].

The CHA2DS2-VASc score has previously been studied in ACS populations. In the study conducted by Trantalis et al., the CHA2DS2-VASc score was shown to have a slightly better predictive power than the GRACE score in assessing cardiovascular prognosis in patients with acute coronary syndrome. Therefore, since the R2CHADS2, R2CHA2DS2-VASc, and R2CHA2DS2-VA scores are independent predictors of in-hospital mortality in our study—superior to conventional CHADS2 and CHA2DS2-VASc—comparing these scores with conventional risk scores may offer insights that could provide significant advantages in future clinical practice [27].

Chronic kidney disease is a well-known independent predictor of adverse cardiovascular outcomes in the context of acute myocardial infarction. Renal dysfunction contributes to a prothrombotic and atherogenic environment which might be increasing vulnerability to ischemic complications and reducing tolerance to procedural interventions, such as p-PCI. The inclusion of renal function into existing scores, as seen in R2CHADS2, R2CHA2DS2-VASc, and R2CHA2DS2-VA, reflects this critical relationship [28,29,30].

The findings of this study hold notable relevance for clinical practice. Incorporating the R2CHADS2, R2CHA2DS2-VASc, and R2CHA2DS2-VA scoring systems into emergency department protocols may assist in the prompt identification of patients at elevated risk upon hospital admission. This early risk recognition could guide clinicians toward more proactive care, such as prioritizing these patients for close monitoring in intensive care settings, initiating aggressive treatment plans, or initiating timely discussions about the patient’s prognosis and care preferences.

Since the sex category has been removed from the conventional CHA2DS2-VASc score, which assesses thromboembolic risk in patients with AF, in the current ESC guideline, studies have been conducted to validate the R2CHA2DS2-VA score—derived from the updated CHA2DS2-VA score—in various patient populations. However, there remains a need for validation studies specifically in STEMI patients [31].

## 5. Limitations

Our study has several limitations. First of all, our study was designed as a single center and retrospective study. Second, long-term follow-up of the study population is lacking. It is unclear whether our results have similar strength regarding long-term mortality. Third, the term “in-hospital mortality” encompasses all deaths. Since the study population consists of STEMI patients, the relationship of the scores with cardiovascular mortality specifically could not be evaluated. While the study emphasized the clinical importance of R2CHADS2, R2CHA2DS2-VASc, and R2CHA2DS2-VA, a direct head-to-head comparison with established ACS-specific tools, like GRACE or TIMI scores, was not performed.

## 6. Conclusions

To the best of our knowledge this is the first clinical study in the literature to evaluate R2CHADS and R2CHA2DS2-VASc scores to predict in-hospital mortality in STEMI patients treated with p-PCI. As simple, cost-effective and reproducible risk scores, R2CHADS and R2CHA2DS2-VASc scores were independent predictors of in-hospital in our study. Their power to predict in-hospital mortality in STEMI patients and non-negligible advantages justify their use in clinical practice.

## Figures and Tables

**Table 1:** Baseline clinical characteristic of the control and in-hospital mortality groups.

Variable	Control Group n = 813	In-Hospital Mortality Group n = 44	*p* Value
Age (years)	57.72 ± 10.96	65.36 ± 12.45	<0.001
Female gender	170 (20.9)	14 (31.8)	0.085
BMI (kg/m^2^)	23.95 ± 7.71	22.34 ± 4.07	0.04
HTN (n, %)	364 (44.7)	23 (52.9)	0.327
DM (n, %)	223 (27.4)	19 (43.2)	0.023
CVA (n, %)	26 (3.2)	3 (6.9)	0.182
EF (%)	46.48 ± 10.53	29.56 ± 9.07	<0.001
SBP (mmHg)	138.03 ± 28.70	118.73 ± 40.98	0.001
DBP (mmHg)	79.03 ± 16.46	68.38 ± 20.71	0.001
Heart rate (bpm)	81.56 ± 19.48	99.70 ± 28.33	<0.001
Albumin (g/dL)	3.82 ± 0.37	3.35 ± 0.53	<0.001
CRP (mg/L)	2.43 ± 3.65	6.20 ± 7.38	0.001
WBC (×10^9^/L)	12.31 ± 4.58	15.83 ± 8.30	0.012
HGB (g/dL)	13.82 ± 1.87	12.50 ± 2.47	<0.001
PLT (×10^9^/L)	238.59 ± 65.04	226.36 ± 74.12	0.295
LDL (mg/dL)	115.18 ± 39.83	116.08 ± 51.55	0.707
Glucose (mg/dL)	160.68 ± 83.85	248.34 ± 160.98	<0.001
Creatinine (mg/dL)	1.02 ± 2.88	2.06 ± 1.96	<0.001
GFR (mL/min)	88.90 ± 23.03	50.18 ± 31.25	<0.001
cTnI (pg/mL)	1902.21 ± 8322.58	2611.07 ± 8726.86	0.002
Pro-BNP (pg/mL)	250.31 ± 387.64	1160.73 ± 1006.33	<0.001
<TIMI3 FLOW	89 (10.9)	11 (25)	0.010
MULTIVESSEL DISEASE	186 (22.9)	16 (36.4)	0.040
CHADS2 score	0.87 ± 0.98	1.47 ± 1.04	<0.001
R2CHADS2 score	1.07 ± 1.28	2.79 ± 1.42	<0.001
CHA2DS2-VASc score	1.36 ± 1.39	2.43 ± 1.56	<0.001
R2CHA2DS2-VASc score	1.56 ± 1.70	3.75 ± 1.81	<0.001
CHA2DS2-VA score	1.15 ± 1.21	2.11 ± 1.41	<0.001
R2CHA2DS2-VA score	1.36 ± 1.54	3.34 ± 1.73	<0.001

(BMI: body mass index; bpm: beats per minute; CHADS2: congestive heart failure, hypertension, age ≥ 75 years, diabetes mellitus, prior stroke or TIA or thromboembolism (2 points); CHA2DS2-VA: congestive heart failure, hypertension, age ≥ 75 years (2 points), diabetes mellitus, prior stroke or TIA or thromboembolism (2 points), vascular disease, age 65 to 74 years; CHA2DS2-VASc: congestive heart failure, hypertension, age ≥ 75 years (2 points), diabetes mellitus, prior stroke or TIA or thromboembolism (2 points), vascular disease, age 65 to 74 years, sex category; CRP: C-reactive protein; cTnI: cardiac troponin-I; CVA: cerebrovascular accident; DBP: diastolic blood pressure; DM: diabetes mellitus; EF: ejection fraction; GFR: glomerular filtration rate; HGB: hemoglobin; HTN: hypertension; LDL: low-density lipoprotein; PLT: platelet; Pro-BNP: pro-B-type natriuretic peptide; R2CHADS2: renal failure (2 points), congestive heart failure, hypertension, age ≥ 75 years, diabetes mellitus, prior stroke or TIA or thromboembolism (2 points); R2CHA2DS2-VA: renal failure (2 points), congestive heart failure, hypertension, age ≥ 75 years (2 points), diabetes mellitus, prior stroke or TIA or thromboembolism (2 points), vascular disease, age 65 to 74 years; R2CHA2DS2-VASc: renal failure (2 points), congestive heart failure, hypertension, age ≥ 75 years (2 points), diabetes mellitus, prior stroke or TIA or thromboembolism (2 points), vascular disease, age 65 to 74 years, sex category; SBP: systolic blood pressure; WBC: white blood cell).

**Table 2:** Univariate analysis of in-hospital mortality.

	OR	95% CI	*p*
Age	1.065	1.035–1.097	<0.001
HTN	0.739	0.402–1.356	0.328
BMI	0.879	0.791–0.978	0.018
DM	2.014	1.088–3.730	0.026
CVA	2.218	0.645–7.630	0.206
EF	0.853	0.817–0.892	<0.001
CHF	16.180	4.184–62.574	<0.001
SBP	0.976	0.965–0.988	<0.001
DBP	0.961	0.941–0.980	<0.001
Heart rate	1.037	1.023–1.050	<0.001
Albumin	0.066	0.030–0.145	<0.001
CRP	1.136	1.081–1.194	<0.001
Glucose	1.006	1.004–1.009	<0.001
GFR	0.953	0.943–0.964	<0.001
<TIMI3 FLOW	0.766	0.268–2.189	0.619
MULTIVESSEL DISEASE	1.929	1.022–3.643	0.053
CHADS2	1.723	1.310–2.265	<0.001
R2CHADS2	2.031	1.674–2.464	<0.001
CHA2DS2-VASc	1.579	1.302–1.917	<0.001
R2CHA2DS2-VASc	1.713	1.473–1.993	<0.001
CHA2DS2-VA	1.695	1.357–2.090	<0.001
R2CHA2DS2-VA	1.811	1.542–2.128	<0.001

(BMI: body mass index; CHADS2: congestive heart failure, hypertension, age ≥ 75 years, diabetes mellitus, prior stroke or TIA or thromboembolism (2 points); CHA2DS2-VASc: congestive heart failure, hypertension, age ≥ 75 years (2 points), diabetes mellitus, prior stroke or TIA or thromboembolism (2 points), vascular disease, age 65 to 74 years, sex category; CHF: congestive heart failure; CRP: C-reactive protein; CVA: cerebrovascular accident; DBP: diastolic blood pressure; DM: diabetes mellitus; EF: ejection fraction; GFR: glomerular filtration rate; HTN: hypertension; R2CHADS2: renal failure (2 points), congestive heart failure, hypertension, age ≥ 75 years, diabetes mellitus, prior stroke or TIA or thromboembolism (2 points); R2CHA2DS2-VASc: renal failure (2 points), congestive heart failure, hypertension, age ≥ 75 years (2 points), diabetes mellitus, prior stroke or TIA or thromboembolism (2 points), vascular disease, age 65 to 74 years, sex category; SBP: systolic blood pressure).

**Table 3:** A: Multivariate logistic regression analysis of CHADS2 and R2CHA2DS2 scores. B: Multivariate logistic regression analysis of CHA2DS2-VASc and R2CHA2DS2-VASc scores. C: Multivariate logistic regression analysis of CHA2DS2-VA and R2CHA2DS2-VA scores.

**A**						
	**Model A for CHADS2**			**Model B for R2CHADS2**		
	**OR**	**95% **CI	***p***	**OR**	**95% CO**	***p***
BMI	0.869	0.707–1.068	0.182	0.858	0.709–1.037	0.114
EF	0.888	0.818–0.964	0.004	0.878	0.812–0.950	0.001
SBP	0.983	0.960–1.006	0.144	0.987	0.966–1.008	0.220
Heart rate	1.015	0.982–1.049	0.375	1.016	0.987–1.046	0.291
Albumin	0.596	0.094–3.797	0.584	0.266	0.053–1.326	0.106
CRP	0.996	0.888–1.118	0.950	1.054	0.949–1.169	0.326
Glucose	1.001	0.996–1.006	0.657	1.000	0.995–1.005	0.935
GFR	0.944	0.912–0.976	0.001			
CHADS2	1.453	0.687–3.072	0.328			
R2CHADS2				2.214	1.381–3.548	0.001
BMI: body mass index; CHADS2: congestive heart failure, hypertension, age ≥ 75 years, diabetes mellitus, prior stroke or TIA or thromboembolism (2 points); CRP: C-reactive protein; EF: ejection fraction; GFR: glomerular filtration rate; R2CHADS2: renal failure (2 points), congestive heart failure, hypertension, age ≥ 75 years, diabetes mellitus, prior stroke or TIA or thromboembolism (2 points); SBP: systolic blood pressure.						
**B**						
	**Model A for CHA2DS2-VASc**			**Model B for R2CHA2DS2-VASc**		
	**OR**	**95% CI**	***p***	**OR**	**95% CO**	***p***
BMI	0.887	0.726–1.084	0.241	0.891	0.743–1.068	0.211
EF	0.982	0.819–0.960	0.003	0.872	0.810–0.940	<0.001
SBP	0.982	0.959–1.004	0.114	0.986	0.966–1.006	0.176
Heart rate	1.014	0.983–1.047	0.380	1.014	0.986–1.043	0.330
Albumin	0.553	0.087–3.529	0.531	0.229	0.043–1.220	0.084
CRP	1.004	0.901–1.119	0.940	1.060	0.959–1.170	0.255
Glucose	1.001	0.995–1.006	0.810	1.001	0.996–1.006	0.664
GFR	0.944	0.912–0.976	0.001			
CHA2DS2-VASc	1.429	0.798–2.559	0.230			
R2CHA2DS2-VASc				1.910	1.302–2.802	0.001
BMI: body mass index; CHA2DS2-VASc: congestive heart failure, hypertension, age ≥ 75 years (2 points), diabetes mellitus, prior stroke or TIA or thromboembolism (2 points), vascular disease, age 65 to 74 years, sex category; CRP: C-reactive protein; EF: ejection fraction; GFR: glomerular filtration rate; R2CHA2DS2-VASc: renal failure (2 points), congestive heart failure, hypertension, age ≥ 75 years (2 points), diabetes mellitus, prior stroke or TIA or thromboembolism (2 points), vascular disease, age 65 to 74 years, sex category; SBP: systolic blood pressure.						
**C**						
	**Model A for CHA2DS2-VA**			**Model B for R2CHA2DS2-VA**		
	**OR**	**95% CI**	***p***	**OR**	**95% CO**	***p***
BMI	0.869	0.710–1.062	0.170	0.864	0.715–1.044	0.130
EF	0.887	0.818–0.961	0.003	0.874	0.810–0.943	0.001
SBP	0.985	0.964–1.006	0.161	0.988	0.969–1.048	0.248
Heart rate	1.017	0.986–1.049	0.298	1.019	0.990–1.048	0.207
Albumin	0.600	0.094–3.806	0.587	0.275	0.052–1.464	0.130
CRP	1.011	0.907–1.127	0.846	1.065	0.960–1.181	0.235
Glucose	1.001	0.997–1.006	0.541	1.002	0.997–1.006	0.460
GFR	0.946	0.916–0.979	0.001			
CHA2DS2-VA	1.411	0.790–2.520	0.245			
R2CHA2DS2-VA				1.979	1.345–2.910	0.001
BMI: body mass index; CHA2DS2-VA: congestive heart failure, hypertension, age ≥ 75 years (2 points), diabetes mellitus, prior stroke or TIA or thromboembolism (2 points), vascular disease, age 65 to 74 years; CRP: C-reactive protein; EF: ejection fraction; GFR: glomerular filtration rate; R2CHA2DS2-VA: renal failure (2 points), congestive heart failure, hypertension, age ≥ 75 years (2 points), diabetes mellitus, prior stroke or TIA or thromboembolism (2 points), vascular disease, age 65 to 74 years; SBP: systolic blood pressure.						

**Table 4:** AUC, cut-off, sensitivity and specificity values of R2CHADS2, R2CHA2DS2-VASc, and R2CHA2DS2-VA scores for prediction of in-hospital mortality.

	AUC	*p*	CI 95%	Cut-Off	Sensitivity	Specificity
R2CHADS2	0.810	<0.001	0.744–0.877	1.5	84.1	71.2
R2CHA2DS2-VASc	0.807	<0.001	0.742–0.872	2.5	79.5	74.7
R2CHA2DS2-VA	0.814	<0.001	0.751–0.878	2.5	70.5	79.7

R2CHADS2: renal failure (2 points), congestive heart failure, hypertension, age ≥ 75 years, diabetes, prior stroke or TIA or thromboembolism (2 points); R2CHA2DS2-VASc: renal failure (2 points), congestive heart failure, hypertension, age ≥ 75 years (2 points), diabetes mellitus, prior stroke or TIA or thromboembolism (2 points), vascular disease, age 65 to 74 years, sex category; R2CHA2DS2-VA: renal failure (2 points), congestive heart failure, hypertension, age ≥ 75 years (2 points), diabetes mellitus, prior stroke or TIA or thromboembolism (2 points), vascular disease, age 65 to 74 years.

**Table 5:** In-hospital mortality rates according to R2CHADS2, R2CHA2DS2-VASc, and R2CHA2DS2-VA scores.

	R2CHA2DS2 ≤ 2 (n = 726)	R2CHA2DS2 > 2 (n = 132)	*p*
In-hospital mortality (n, %)	18 (2.5)	26 (19.7)	<0.001
	**R2CHA2DS2-VASc ≤ 3** (n = 722)	**R2CHA2DS2-VASc > 3** (n = 136)	
In-hospital mortality (n, %)	20 (2.8)	24 (17.6)	<0.001
In-hospital mortality (n, %)	**R2CHA2DS2-VA ≤ 3** (n = 753)	**R2CHA2DS2-VA > 3** (n = 105)	
	23 (3.1)	21 (20)	<0.001

CHA2DS2-VASc: congestive heart failure, hypertension, age ≥ 75 years (2 points), diabetes mellitus, prior stroke or TIA or thromboembolism (2 points), vascular disease, age 65 to 74 years, sex category; R2CHA2DS2-VASc: renal failure (2 points), congestive heart failure, hypertension, age ≥ 75 years (2 points), diabetes mellitus, prior stroke or TIA or thromboembolism (2 points), vascular disease, age 65 to 74 years, sex category; R2CHA2DS2-VA: renal failure (2 points), congestive heart failure, hypertension, age ≥ 75 years (2 points), diabetes mellitus, prior stroke or TIA or thromboembolism (2 points), vascular disease, age 65 to 74 years.

**Table 6:** Calibration of R2CHADS2, R2CHA2DS2-VASc, and R2CHA2DS2-VA scores.

Group	Upper Boundaries of Predicted Probabilities	Observed Deaths	Expected Deaths	Observed Alvie	Expected Alive	Total Observations	*p*
R2CHADS2							
1	0.028	4	5.1	354	352.9	358	0.159
2	0.056	3	6.6	225	221.4	228	
3	0.057	11	7.9	128	131.1	139	
4	0.505	26	24.4	106	107.6	132	
R2CHA2DS2-VASc							
1	0.013	3	3.8	287	286.2	290	0.188
2	0.022	2	4.6	201	198.4	203	
3	0.038	4	4.7	120	119.3	124	
4	0.063	11	6.7	94	98.3	105	
5	0.501	24	24.1	112	111.9	136	
R2CHA2DS2-VA							
1	0.024	3	4.3	312	310.7	315	0.187
2	0.043	2	5.2	211	207.8	213	
3	0.038	8	5.8	126	128.2	134	
4	0.076	10	6.9	81	84.1	91	
5	0.469	21	21.7	84	83.3	105	

## Data Availability

The original data presented in the study are openly available in an Excel file.
